# Manual Ability Classification System (MACS): reliability between therapists and parents in Brazil

**DOI:** 10.1590/bjpt-rbf.2014.0065

**Published:** 2015

**Authors:** Daniela B. R. Silva, Carolina A. R. Funayama, Luzia I. Pfeifer

**Affiliations:** 1 Divisão de Terapia Ocupacional, Departamento de Neurociências e Ciências do Comportamento, Faculdade de Medicina de Ribeirão Preto (FMRP) Universidade de São Paulo (USP), Ribeirão Preto, SP, Brazil; 2 Departamento de Neurociências e Ciências do Comportamento, FMRP, USP, Ribeirão Preto, SP, Brazil

**Keywords:** cerebral palsy, MACS, reproducibility of results, rehabilitation

## Abstract

**BACKGROUND:**

The Manual Ability Classification System (MACS) has been widely used to describe the manual ability of children with cerebral palsy (CP); however its reliability has not been verified in Brazil.

**OBJECTIVE:**

To establish the inter- and intra-rater reliability of the Portuguese-Brazil version of the MACS by comparing the classifications given by therapists and parents of children with CP.

**METHOD:**

Data were obtained from 90 children with CP between the ages of 4 and 18 years, who were treated at the neurology and rehabilitation clinics of a Brazilian hospital. Therapists (an occupational therapist and a student) classified manual ability (MACS) through direct observation and information provided by parents. Therapists and parents used the Portuguese-Brazil version of the MACS. Intra- and inter-rater reliability was obtained using unweighted Kappa coefficient (k) and intra-class correlation coefficient (ICC). The Chi-square test was used to identify the predominance of disagreements in the classification of parents and therapists.

**RESULTS:**

An almost perfect agreement resulted among therapists [K=0.90 (95% CI 0.83-0.97); ICC=0.97 (95%CI 0.96-0.98)], as well as with intra-rater (therapists), with Kappa ranging between 0.83 and 0.95 and ICC between 0.96 and 0.99 for the evaluator with more and less experience in rehabilitation, respectively. The agreement between therapists and parents was fair [K=0.36 (95% CI 0.22-0.50); ICC=0.79 (95% CI 0.70-0.86)].

**CONCLUSIONS:**

The Portuguese version of the MACS is a reliable instrument to be used jointly by parents and therapists.

## Introduction

Cerebral palsy (CP) can affect body structures, such as the hands and their components (muscles, joints, bones), as well as several body functions^1^. Hand functions are frequently impaired, resulting in diminished mobility, reduction in muscle strength, lack of control in rapid coordinated movements, presence of involuntary movements^1^, spasticity^1^^,^ 2, and poor postural control^2^. Hand sensory impairments may be observed, involving tactile pressure detection, tactile spatial resolution, and recognition of common objects and shapes^1^. CP may also limit the child's ability to perform functional daily tasks, such as eating, drinking, grooming, dressing, and performing school tasks^1^.

Until recently, most classification systems related to the use of upper limbs in children with cerebral palsy focused on manual function (e.g. House Classification^3^, Modified House Classification^4^, and Zancolli Classification^5^ systems) or on manual functional capacity (e.g. Bimanual Fine Motor Function classification system^6^). In 2006, the new Manual Ability Classification System (MACS) was developed, taking into account the usual performance of children and young people at home, at school, and in the community, instead of focusing on what they are capable of doing best (capacity) in relation to manual ability^7^^,^ 8. This classification system complies with the International Classification of Functioning, Disability and Health^9^.

The MACS aims to classify how children with CP use their hands when handling objects in their daily activities^7^ and the impact that these environmental and personal factors (e.g. motivation and cognition^7^) may have on the children's performance^10^. MACS levels are based on children's ability to initiate handling objects by themselves (eating, dressing, playing, drawing or writing^7^) and their need for assistance or adaptation to perform manual daily life activities that are appropriate for their age. The MACS covers the age group between 4 and 18 years in levels ranging from I to V. The children at level I are able to handle objects easily, those at level II handle most objects but with a little reduced quality or speed, and those at level III handle objects with difficulty and need help to prepare or change activities. At level IV, children handle a limited quantity of objects and require continuous support to partially conclude the activities, and at level V, the children do not handle objects^7^.

The MACS has received international recognition^11^ and has been used in conjunction with the Gross Motor Function Classification System (GMFCS)^12^ to classify children with cerebral palsy. The MACS has been translated into 25 languages as it is recognized as a valid and reliable system to classify the manual ability of children with cerebral palsy^7^^,^ 8 ^,^ 13 ^-^ 18.

Through the use of classification systems like the MACS, it is possible to have a broad vision of the children's overall ability to handle everyday objects, which constitutes an important complement to the diagnosis^19^. The combination of the information provided by this classification and the medical diagnosis makes it possible to define subgroups within the heterogeneous group of cerebral palsy^20^. In clinical practice, it is important to improve communication between families and therapists^20^, helping guide therapeutic objectives and treatment priorities^18^^,^ 21. However, the MACS should not be used as an outcome measure to document the impact of intervention since the classifications, although stable over time^11^, do not have appropriate sensitivity.

Researchers who developed the MACS mentioned that, to obtain information concerning the manner in which the children handle various objects in daily life, it is necessary to ask someone who knows them well^7^. Studies have shown from good to excellent reliability of the MACS when used by different healthcare professionals^7^^,^ 8 ^,^ 10 ^,^ 13 ^,^ 14 ^,^ 16 ^,^ 17 and parents^7^^,^ 15 ^-^ 17.

In Brazil, the MACS was translated and culturally adapted to Portuguese-Brazil according to the procedures suggested by Beaton et al.^22^ and is available for download (http://www.macs.nu); however, the reliability of this classification system has not been established in the country. The objective of the study was: (1) to determine inter and intra-rater (therapist and student) reliability of the Portuguese-Brazil version of the MACS; (2) to compare the classification of therapists and parents in relation to the MACS.

## Method

This is a methodological study aimed at examining the psychometric properties of a classification system. The research was approved by the Research Ethics Committee of Hospital das Clínicas at Faculdade de Medicina de Ribeirão Preto, Universidade de São Paulo (HCFMRP-USP), Ribeirão Preto, SP, Brazil. Parents signed an informed consent form (HCRP no. 12469/2008).

### Participants

To assess the reliability of the MACS, ninety children with CP, aged between four and 18 years, were included. The children were treated at the Occupational Therapy or Physical Therapy Service of the Rehabilitation Center or at the Neurology Outpatient Clinic of HCFMRP-USP between September 2010 and October 2011.

This was a convenience sample and the inclusion criteria were: to be diagnosed with CP, regardless of the type or severity of the motor disability, to be aged between 4 and 18 years, and to understand simple commands. The exclusion criteria were low vision or blindness and/or epilepsy.

### Data collection procedure

Two evaluators collected data simultaneously at separate locations. Evaluator 1 (E1) was an undergraduate occupational therapy student and Evaluator 2 (E2) had nine years of experience in the rehabilitation of children with CP (occupational therapist). None of them were familiar with the MACS and both received training before the start of data collection through an instructional video produced by the authors of the classification. They also classified 10 children who were not included in the research sample. Any questions concerning the selection of the MACS level were clarified with Dr. Eliasson by email. A research assistant (E3), also an occupational therapy student, was responsible for filming each child while handling objects, as well as for filming the parents' report about how their children commonly used their hands to manipulate objects in their daily activities as described below. E3 was also responsible for the selection of videos for the intra-rater reliability test.

E1 observed each child performing the following tasks: 1) Opening a puzzle box or animal memory game box; 2) Emptying the contents of the box onto the table; 3) Handling the pieces of a puzzle or memory game; 4) Placing the pieces back in the box; 5) Closing and opening a zipper; 6) Buttoning and unbuttoning a jacket; 7) Using a spoon to scoop up food (e.g. yogurt or soft cake); 8) Drinking from a cup; 9) Building a tower with 5 blocks; 10) Pounding pegs using a toy hammer; 11) Performing a graphic activity; 12) Putting marbles and/or beads into a jar. After observing the child, E1 also obtained additional information from the parents concerning the typical performance of the children at home and school, in relation to the use of their hands, such as playing, feeding, and using materials at school. E1 classified each child, considering the observation of his/her performance, in combination with the information collected from the parents about the child's use of their hands in their performance of daily activities; this was the first assessment of Evaluator 1 (A1E1). Each child's task performance and the parents' report were filmed by E3 to be used in inter- and intra-rater reliability tests.

While E1 observed the child performing the tasks, E2 read the Portuguese-Brazil version of the MACS to the parents, who had to select a single level of the MACS to classify their child. E2 informed the parents that, in order to select the level of the MACS, it was necessary to consider the manual ability of their children when playing, drawing, writing, and handling objects during feeding and dressing. When parents provided examples of the manual ability of their children, it was reinforced that they should select the level that best described the manual ability. If they were unsure about which level they should select, the difference between the two levels was read to them. E2 simply wrote down the MACS level that the parents selected without any clarification.

Two weeks later, in an attempt to minimize memory bias (i.e. remembering the classification level chosen by parents), E2 classified the children according to the MACS levels by analyzing the videos of the children's observation and the parents' information about the children's performance in daily activities, which was the first assessment of Evaluator 2 (A1E2).

The second assessment (intra-rater reliability) took place one month after the first one. Both evaluators (E1 and E2) watched the videos separately in order to classify the children's manual ability level. This stage included 30 videos selected by E3 (corresponding to 33% of the sample) from various age groups and MACS levels based on the initial classification of the E2.

Parent and therapist classifications were compared using the first assessment performed by E2 (A1E2).

### Statistical analysis

The MACS is an ordinal scale with five levels. Descriptive statistics were used to characterize the participants. Ordinal data were analyzed using percentage agreement and Cohen's unweighted Kappa (k) to examine parent-therapist agreement and intra- and inter-rater agreements. By using the Intra-class Correlation Coefficient (ICC) it was possible to make comparisons with other studies.

The following criteria were used for the Kappa coefficient interpretation: values below zero (poor), between 0.00 and 0.20 (slight), between 0.21 and 0.40 (fair), between 0.41 and 0.60 (moderate), between 0.61 and 0.80 (substantial), and between 0.81 and 1.0 (almost perfect agreement)^23^. The Chi-square test for adherence was used to verify the associations between the perception of parents and therapists in relation to the manual ability classification of children using the MACS.

## Results

The average age of the children was 7.58 years, ranging from 4 to 17.91 years of age. Most were diagnosed as children with spastic bilateral CP (n=61), followed by unilateral spastic (n=17), dyskinetic (n=9), and ataxic (n=3) CP. Regarding gender, the sample was equally distributed.

The children with unilateral CP were classified by the occupational therapist (E2) as MACS levels I or II (35% in level I and 65% in level II). Most of those with bilateral CP were classified as level I (31.1%), although the other children from this group were distributed over the other MACS levels (Table 1). In relation to the parents, the MACS classification was performed mostly by mothers (91%). Table 2 shows additional information about the parents.

**Table 1:** Classifications based on MACS levels performed by the E2 (A1E2) according to the type of cerebral palsy (CP).

CP type	Level I	Level II	Level III	Level IV	Level V	Total
Bilateral spastic	19	13	13	8	8	61
Unilateral spastic	6	11	-	-	-	17
Dyskinetic	-	1	1	3	4	9
Ataxic	-	1	2	-	-	3

**Table 2:** Caregivers' characteristics.

Caregivers’ characteristics	Average	Standard deviation	Minimum	Maximum
Age	34.06	8.77	19	56
Years of schooling	8.80	3.29	0	15
Income^*^	1222.81	787.34	250.00	6000.00
Occupation	(n)			
Unemployed	62			
Employed	28			

### Inter-rater reliability

Table 3 presents the results of inter-rater agreement, considered to be almost perfect [K=0.90, (95% CI 0.83-0.97); ICC=0.98 (95% CI 0.96-0.98)].

**Table 3:** Inter-rater agreement and disagreements (A1E1 and A1 E2).

Evaluator 1	Evaluator 2					
	Level I	Level II	Level III	Level IV	Level V	Total
Level I	24	2^*^	-	-	-	26
Level II	-	23	-	-	-	23
Level III	-	1^*^	15	1^*^	-	17
Level IV	-	-	2^*^	9	-	11
Level V	-	-	-	1^*^	12	13
Total	26	23	17	11	13	90

It can be noted that there were seven disagreements that were not predominant at specific levels (χ^2^=1.57; p=0.67), two of them between levels I and II, one between II and III, three between levels III and IV, and one between levels IV and V.

### Intra-rater reliability

The evaluators (E1 and E2) classified the manual ability of 30 children with cerebral palsy in the initial assessment and after one month. The results show almost perfect agreement between both evaluators. E1 had two disagreements between levels I and II and two others between levels III and IV [K=0.83 (95% CI 0.68-0.98); ICC=0.96, (95% CI 0.68-0.98)]. E2 (the researcher with the most experience in the neurology field) had only one disagreement between levels I and II of the MACS [K=0.95 (95% CI 0.88-1.00); ICC=0.99 (95% CI 0.98-0.99)].

### Reliability between therapists and parents

Fair agreement was observed between the classification performed by the occupational therapist (E2) and the classification performed by the parents [K=0.36 (95% CI 0.22-0.50); ICC=0.79 (95% CI 0.70-0.86)]. Table 4 presents the frequency of agreement and disagreement between E2 and the parents, according to the MACS levels.

**Table 4:** Frequency of agreement and disagreements of the first assessment by evaluator 2 (A1E2) and the parents.

Evaluator 2	MACS Parents					
	Level I	Level II	Level III	Level IV	Level V	Total
Level I	11	13^*^	-	-	-	24
Level II	8^*^	14	4^*^	-	-	26
Level III	2^*^	5^*^	4	6^*^	-	17
Level IV	2^*^	-	1^*^	8	-	11
Level V	-	-	-	3^*^	9	12
Total	23	32	9	17	9	90

* inter-rater disagreements.

It is possible to verify that there were 46 classification agreements between parents and the therapist (E2) and 44 disagreements. Of these, 21 occurred between levels I and II, 9 between levels II and III, 7 between levels III and IV, and 3 between levels IV and V. There were also four disagreements in more than one level between therapist and parents, two of them between levels I and III and the others between levels I and IV. It can be noted through the chi-square test for adherence that the disagreements between levels I and II are the most predominant (χ^2^=36.18; p=0.001).

## Discussion

This study sought to assess the reliability between therapists (occupational therapist and undergraduate occupational therapy student) by means of inter- and intra-rater reliability tests concerning the MACS, with an almost perfect reliability (ICC=0.97; K=0.90). Similar results were found in the validation study of the MACS in Sweden^7^, Korea^16^, Turkey^17^, and Iran^18^, with ICC ranging from 0.95 to 0.98, and in the Netherlands^10^ (K=0.86). These studies involved different health professionals (occupational therapists, physical therapists, physiatrists, and pediatricians).

In this study, video analysis was used for the children's performance while handling objects and the parents' reports was used for the evaluators' classification. Most studies concerning the MACS reliability verified by healthcare professionals have used the direct observation of the children in the clinical context and have shown positive results for the age group proposed by the MACS (4 and 18 years of age)^10^^,^ 13 ^,^ 16 ^,^ 17, especially when additional information about the typical performance was obtained from the parents^16^^,^ 17. The parents observe the children's performance in different environments and in different activities throughout the day, while the professionals observe a limited selection of daily activities^20^ in a clinical environment or school^24^. For that reason, it is important to consider the information of both and also because manual ability is the result of contextual, environmental, and personal factors such as motivation and cognitive ability^7^ and it can vary according to the objects being handled, time of day, and setting^13^. We believe that the information obtained from the parents in this study, combined with the observation of manual ability, resulted in the near perfect agreement between the evaluators, despite their different levels of expertise in the field of neuropediatrics (occupational therapist and student).

The intra-rater reliability was considered almost perfect for both evaluators, with only one disagreement for E2 and four disagreements for E1, who had less experience. Only three studies were found in the literature involving intra-rater reliability (healthcare professionals) of the MACS as part of the process of cultural adaptation and reliability and they were conducted in Korea^16^, Turkey^17^, and Iran^18^, with almost perfect agreement in all of them (ICC ranging from 0.97 and 0.98). In these studies, the healthcare professionals repeated the procedures for the first MACS assessment, which included direct observation and interviewing the parents^16^^,^ 17.

In our research, we used a similar method (direct observation of the child and parent interview), but the second assessment to evaluate intra-rater reliability was made by watching the video recordings of the initial assessment. This procedure has been used in other studies to evaluate intra-rater reliability^25^^,^ 26.

Regarding the reliability between therapists and parents, there was substantial agreement, with disagreements predominantly between levels I and II. The results obtained in this research are similar to Morris et al.^13^, who also found that disagreements between levels I and II were more frequent (K=0.38). The data collection in that study involved direct observation of the children by physical therapists and parent classifications performed using a MACS booklet received by mail, without the possibility of clarifying any questions with the therapist.

In our research, the evaluator simply read the MACS booklet to the parents. If they provided examples of the children's handling of the objects at home and asked whether those examples corresponded to a certain MACS level, the evaluator provided no response so as not to influence the parents, but instead emphasized the need for them to choose one of the levels that was closer to the manual ability of the children, and repeated the levels and/or topics, differentiating the levels if necessary.

Among the studies that had higher rates of agreement, parents and physical therapists read the MACS booklet and the parents' questions concerning the classification were answered by the researcher^7^^,^ 17 or the therapist in charge^16^.

It should be acknowledged that, in its descriptions, the MACS uses terms like "quality", "speed", and "independence", which are more commonly used by healthcare professionals in their clinical practice and which can be interpreted differently by parents and therapists. In addition, these terms have to be considered in relation to the child's age^20^, since there are differences between the objects that a four-year-old is capable of handling and those an adolescent is able to handle, such as fragile and sharp objects^7^, as well as the level of independence, which can be very complex for the parents to analyze. The authors of the MACS recognize that additional instructions, examples of daily activity performance, and information such as an instructional video would probably make the process of classification easier for the parents^20^.

Other aspects that can be related to disagreements between therapists and parents' classification is that healthcare professionals, when using classifications like the MACS, tend to evaluate the children in clinical environments, which are designed to allow their optimal functioning and minimize the negative effects of the environment^24^, while parents consider a broader range of environments, such as home, school, and community, when classifying their child's performance^24^^,^ 27 ^,^ 28. Although the evaluators (E1 and E2) also considered the parents' report of the children's typical manual ability performance in this study, they did not observe the children in non-therapeutic environments and, even if they did, their view might differ from the parents', as the latter adopt a perspective relevant to the activities performed over a whole day, while therapists almost inevitably take into account a limited selection of structured daily activities^20^. Therefore, it is understandable that parents and therapists perceive the children's manual ability differently, as the latter often have limited information about the children's performance in the different environments they attend.

However, taking into consideration the limitations of the MACS as mentioned, such as the use of terminology directed to healthcare professionals and the complexity of the manual ability concept, it is suggested that therapists provide information to the parents about the concepts present in the MACS, examples concerning the terminology used, and that they answer their questions to assist with the classification. In addition, parents and therapists should be able to jointly classify the manual ability of children with CP, as the thinking process conducted by the parents in relation to their children's manual ability and the communication of information to healthcare professionals can provide better personal understanding and improve self-confidence^24^, in addition to providing a starting point for decision-making and intervention planning for the children^29^.

Within the limitations of the study is the fact that the classification of the MACS was done by parents based only on the reading of the MACS booklet and no explanation about specific terms or clarification of questions was provided by the researcher. Further studies about content validation and stability of the MACS by therapists and parents are needed, as well as the comparison of the classification by therapists and parents who had or had not been trained through the institutional MACS video.

Therefore, we conclude that the MACS has been shown to be a reliable classification that can be used by health professionals with different levels of experience and by parents. Even if therapists and parents perceive the manual ability of children with CP differently, the information from both sources should be complementary. The use of the MACS in clinical practice can be an important starting point to set intervention targets for the children in partnership with the family. In addition, the health professionals should give examples/information about manual ability and clarify the parents' questions about the description of the MACS levels in order to enhance their understanding of the classification.
